# Retrospective analysis of the standardized BARD criteria for acute cholangitis in biliary atresia patients

**DOI:** 10.1002/jpr3.12071

**Published:** 2024-04-12

**Authors:** Omid Madadi‐Sanjani, Ana M. Calinescu, Nathalie Rock, Valerie A. McLin, Marie Uecker, Joachim F. Kuebler, Claus Petersen, Barbara E. Wildhaber

**Affiliations:** ^1^ Department of Pediatric Surgery Hannover Medical School Hannover Germany; ^2^ Swiss Pediatric Liver Center Geneva University Hospitals Geneva Switzerland; ^3^ Department of Pediatrics, Gynecology, and Obstetrics, Division of Child and Adolescent Surgery Geneva University Hospitals Geneva Switzerland; ^4^ Department of Pediatrics, Gynecology and Obstetrics University of Geneva Geneva Switzerland; ^5^ Department of Pediatrics, Gynecology, and Obstetrics, Gastroenterology, Hepatology and Nutrition Unit, Division of Pediatric Specialties Geneva University Hospitals Geneva Switzerland

**Keywords:** clinical application, hepatoportoenterostomy, Kasai, outcome

## Abstract

**Objectives:**

In 2022, the Biliary Atresia and Related Diseases (BARD) community reached a consensus for the definition of suspected and confirmed cholangitis for biliary atresia (BA) patients after hepatoportoenterostomy (HPE). This study assessed the new standardized BARD definition in a retrospective, multicenter cohort study.

**Methods:**

We included BA cases managed between 2010 and 2020 at the Hannover Medical School and Geneva University Hospitals' Swiss Pediatric Liver Center. The standardized BARD cholangitis definition assesses four clinical items and four imaging/laboratory items to define cholangitis. The definition was retrospectively applied to all BA cases having presented, according to their physician, cholangitis within the first year after the HPE. The diagnosis defined by the standardized BARD definition was compared with the final clinical diagnosis made by physicians. The Spearman's correlation coefficient was used to test for correlation between diagnoses made by standardized and clinical appreciation.

**Results:**

Of 185 consecutive BA patients, 59 (32%) had at least one episode of cholangitis within the first year after HPE. The correlation between the clinician's impression and the standardized BARD definition was very strong (*r* = 0.8). Confirmed cholangitis definition coincided with the clinician's impression (2.5 [±0.7]/4 clinical items, 2.6 [±0.5]/4 imaging/laboratory items). For suspected cholangitis, the threshold for diagnosis was lower within the standardized BARD definition (1.1 [±0.3]/4 clinical items, 2.2 [±0.8]/4 laboratory/imaging items).

**Conclusions:**

This first retrospective application of the standardized BARD cholangitis definition reveals a very strong correlation with the physician's assessment before standardization. A prospective study is needed to further refine the standardized definition for cholangitis in BA patients.

## Abstract (highlights)

**What is Known:**

- Acute cholangitis is a life‐threatening diagnosis in biliary atresia (BA) patients.
- The definition for acute cholangitis after Kasai hepatoportoenterostomy in BA patients is physician‐dependent.

**What is New:**

- The standardized Biliary atresia and Related Diseases (BARD) cholangitis definition correlates very strongly with the physician's clinical judgment.
- The standardized BARD cholangitis definition may be used to evaluate treatment response in acute cholangitis and to distinguish cholangitis from the natural course of the disease.

## 1 INTRODUCTION

Acute cholangitis is the most common complication after Kasai hepatoportoenterostomy (HPE) in biliary atresia (BA) patients.^1^ Recurrent cholangitis episodes impact patients' prognosis, mainly overall and native liver survival, by speeding up the cirrhosis process^2^, ^3^ and thus the need for liver transplantation.^4^, ^5^, ^6^, ^7^, ^8^, ^9^, ^10^ Therefore, decreasing cholangitis rates and/or optimizing cholangitis management is paramount for BA patients after HPE.

Attempting to compare BA associated cholangitis rates, prognostic factors, and management strategies in the current body of literature is a challenging task. The lack of a consistent or universally accepted definition of BA associated cholangitis post‐HPE, together with the wide range of prophylactic practices for cholangitis, are two major impediments to generalize recommendations and practices.^11^

For this reason, a standardized definition for suspected and confirmed BA associated cholangitis after HPE was recently developed by the Biliary Atresia and Related Diseases (BARD) community.^11^ The role of this standardized definition for BA cholangitis after HPE is (i) to provide a framework for diagnostic criteria (clinical, imaging, and laboratory) for cholangitis and (ii) to enable comparisons and patient inclusion in clinical studies.

The primary aim of the current study was to retrospectively assess the correlation between the standardized BARD cholangitis definition and expert clinical judgment in BA patients in two centers, the Hannover Medical School and Geneva University Hospitals' Swiss Pediatric Liver Center. Secondary objectives were (i) to investigate in detail the use of the standardized BARD different clinical, imaging, and laboratory elements and (ii) to assess the standardized BARD cholangitis definition before and after treatment implementation. We hypothesized that there is a strong correlation between the clinician's impression of cholangitis and the standardized BARD cholangitis definition.

## 2 METHODS

**Figure 1:**
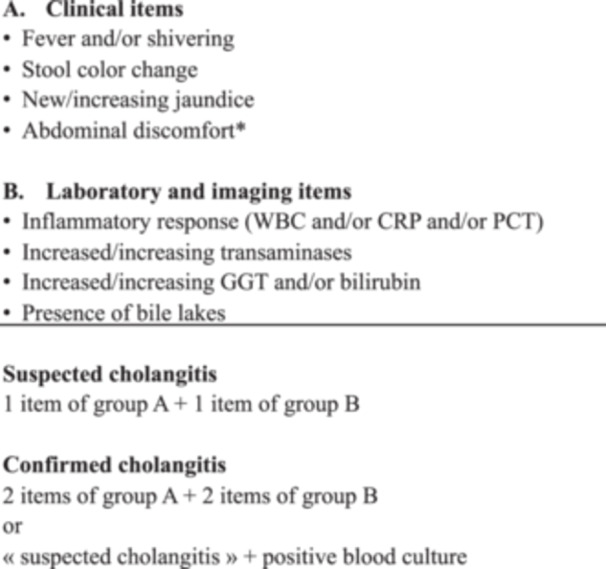
The Standardized Biliary Atresia and Related Diseases (BARD) cholangitis guidelines for the diagnosis of suspected and confirmed cholangitis within the first year following hepatoportoenterostomy for biliary atresia. *Vomiting, poor feeding, irritability. CRP, C‐reactive protein; GGT, gamma‐glutamyl transpeptidase; PCT, procalcitonin; WBC, white blood count.

All consecutive patients with BA having undergone HPE between 2010 and 2020 at the Hannover Medical School and Geneva University Hospitals' Swiss Pediatric Liver Center were included. Patients not having had a clinical diagnosis of acute cholangitis and BA patients without a HPE were excluded. For the excluded patients with HPE, the standardized BARD cholangitis definition was retrospectively applied at every follow‐up visit (1 month, 6 months, and 1 year), not to miss falsely excluded patients. Charts of all BA patients diagnosed by their physician with acute cholangitis during the first year after HPE were analyzed. The study approach was to consider criteria as positive if explicitly met during index admission and if not met, they were considered as negative. Acute cholangitis is referred to as cholangitis. The standardized BARD definition (Figure 1) was retrospectively applied to the cohort of patients having presented a first episode of suspected and/or confirmed cholangitis according to the physicians' judgment. The standardized diagnosis was then compared with the final clinical diagnosis made by physicians. The application of the standardized BARD cholangitis definition was performed pre‐ and posttreatment. Further, to test for the accuracy of the standardized definition, the number of the different clinical items (group A, see below) and imaging and laboratory items (group B, see below) were analyzed in detail.

### 2.1 Standardized BARD criteria for cholangitis within the first year following HPE

In 2021/22, the BARD network elaborated a standardized definition for the first episode of cholangitis within the first year following HPE.^11^ In summary, clinical items (group A) and imaging and laboratory items (group B) are used in number(s) and combination(s) to lead to the standardized diagnosis of either suspected or confirmed cholangitis (Figure 1). Fever was defined as body temperature >38°. Shivering was considered positive if the terms “shaking chills,” “chills,” or “shivers” were identified during the index admission.

The stool color change and new/increasing jaundice relied on admitting doctor's notes or parental history. Thresholds for declaring a positive laboratory test were set at the upper limit of normal for the tests *or* at the “baseline” values recorded during the previous patient appointments.

### 2.2 HPE procedure and postoperative care

All HPE procedures were performed by a designated team of pediatric hepatobiliary surgeons at both centers via laparotomy and pre‐incisional liver eversion. Intraoperative liver biopsies were performed. There was no postoperative abdominal drainage. All patients were transferred postoperatively to the intensive care unit for at least 24 h. Postoperative enteral feedings were started between postoperative Days 1 and 3. A central line was placed during the HPE procedure which was then used for antibiotic treatment as detailed below. On the pediatric surgery wards, daily weight, temperature, and stool color were documented.

### 2.3 Post‐HPE management

The postoperative treatment protocol included administration of UDCA and fat‐soluble vitamins. In addition, patients in Hannover received a 2‐week course of intravenous antibiotics, followed by oral prophylaxis for 6 months. Patients in Geneva received a 7‐day course of intravenous antibiotics.

Hannover Medical School used adjuvant steroid therapy (2 mg daily budesonide administered rectally 4−7 days after HPE and continued for 3 months) since 2011, while Geneva University Hospitals discontinued steroid use in 2011.

Patients were regularly and closely followed‐up in the Swiss Pediatric Liver Center and Hannover Medical School Liver Unit. If patients returned to their local hospitals, the local healthcare providers reliably reported any unexpected event to the respective pediatric liver units.

### 2.4 BA associated cholangitis diagnosis

During the study period, physicians based the initial diagnosis of cholangitis on changes in the liver tests and inflammatory parameters (i.e., liver transaminases, gamma‐glutamyl transpeptidase [GGT], bilirubin, leukocytes, C‐reactive protein, procalcitonin), as well as clinical symptoms including fever, onset/increase of jaundice and acholic stools, after initial postoperative colored stool without any ponderation for the different parameters. In the case of fever and/or increase of inflammatory parameters with changes in liver function tests, other sources of infection were ruled out, including urine and blood and chest X‐ray if deemed necessary.

Since all patients were diagnosed before the standardized BARD cholangitis definition was published, physicians were not influenced by the standardized BARD definition. As there was no gold standard for the definition of acute cholangitis in patients with BA after HPE to date, the final clinical diagnosis made by physicians was considered to be the final. The diagnosis initially made by physicians did not distinguish between suspected or confirmed cholangitis.

### 2.5 Antibiotic treatment for acute cholangitis

For the Hannover Medical School, three antibiotic treatment protocols were used during the study period: intravenous piperacillin/tazobactam, intravenous cefotaxime, or intravenous meropenem for 2−3 weeks, per physician discretion.

For the Geneva University Hospitals, piperacillin/tazobactam was used throughout the observation period for 3 weeks.

### 2.6 Statistical analysis

Data are displayed as mean and standard deviation. Statistical analysis was performed with *t*‐tests using GraphPad Prism software version 6.0 (GraphPad Software). *p* < 0.05 was considered statistically significant. Spearman's correlation coefficient was used to test for correlation between clinical and standardized definition; *r* > 0.4 and <0.6 was considered a moderate association, *r* > 0.6 and <0.8 was considered a strong association, and *r* > 0.8 and <1 as very strong correlation. Cohen's Kappa (K) was used to assess the interrater agreement for the physician‐based diagnosis of cholangitis and the diagnosis according to the BARD definition (GraphPad). Based on the Altman scheme, the strength of agreement was interpreted as very good for a *K* > 0.8.

## 3 RESULTS

Of 185 consecutive BA patients, 59 (32%) experienced at least one episode of cholangitis within the first year after the HPE, diagnosed by their physician. Table 1 summarizes descriptive data of these patients. Fifty‐three(53) patients (90%) were treated with intravenous piperacillin/tazobactam monotherapy after diagnosis, while cefotaxime was used twice (3%), meropenem monotherapy once (2%), and a combined treatment of meropenem with vancomycin, ceftriaxone or piperacillin, and tazobactam in three patients (5%).

**Table 1:** Characteristics of biliary atresia patients with cholangitis within the first year after HPE, showing their clinical items (group A) and laboratory and imaging items (group B) at the time of cholangitis diagnosis.

Patient characteristics (*n* = 59)	
Male:female ratio	1: 1.5
Age at HPE (days)	59 ± 20
Post‐HPE steroid treatment	*n* = 37 (63%)
Elements at cholangitis diagnosis as defined by physicians	
Clinical items (group A)	
Fever and/or shivering	*n* = 24 (41%)
Stool color change	*n* = 19 (32%)
New/increasing jaundice	*n* = 12 (20%)
Abdominal discomfort^a^	*n* = 16 (27%)
Laboratory and imaging items (group B)	
Inflammatory response (WBC and/or CRP and/or PCT)	*n* = 41 (70%)
Increased/increasing transaminases	*n* = 40 (68%)
Increased/increasing GGT and/or bilirubin	*n* = 53 (90%)
Presence of intrahepatic bile lakes	*n* = 1 (2%)
Positive blood cultures	*n* = 1 (2%)

Abbreviations: CRP, C‐reactive protein; GGT, gamma‐glutamyl transpeptidase; HPE, hepatoportoenterostomy; PCT, procalcitonin; WBC, white blood count.

^a^ Vomiting, poor feeding, irritability.

### 3.1 Retrospective application of the standardized BARD cholangitis definition

Of the 59 BA patients diagnosed with a first cholangitis episode by physicians within the first year after HPE, 44 (75%) matched the standardized BARD cholangitis definition (suspected and confirmed). Of those 44 patients, 30 (68%) had, according to the standardized definition, suspected cholangitis, whilst 14 (32%) had confirmed cholangitis. Fifteen patients with clinical cholangitis diagnoses (15/59; 25%) did not meet the criteria for cholangitis according to the standardized BARD definition. The Cohen's Kappa for the overall cholangitis diagnosis based on the clinician's appreciation and using the standardized BARD guidelines (suspected and confirmed) showed very good agreement with a Kappa of 0.803 (95% confidence interval [CI]: 0.709–0.896).

None of the excluded patients had a suspected or confirmed cholangitis when retrospectively applying the standardized BARD definition.

### 3.2 Clinical elements for the standardized BARD cholangitis definition

Clinical symptoms (group A) were present in 46/59 (78%) children at cholangitis diagnosis. Details are summarized in Table 1. Upon stratification of the clinical symptoms according to the standardized BARD cholangitis definition, confirmed cholangitis patients per definition had 2.5 (±0.7) items at diagnosis, for suspected cholangitis 1.1 (±0.3) items, and patients clinically diagnosed with acute cholangitis but not complying with the standardized BARD acute cholangitis definition had 0.3 (±0.7) items (Table 2).

**Table 2:** Clinical, laboratory, and imaging items documented pre‐ and post‐antibiotic treatment for cholangitis, stratified into *confirmed* and *suspected* cholangitis, as well as *not confirmed diagnosis*, according to the standardized BARD definition.

Cholangitis according to the standardized BARD criteria	Pretreatment elements	Posttreatment elements	*p* Value
Confirmed cholangitis (*n* = 14)			
Clinical items	2.5 ± 0.7	0.2 ± 0.4	<0.0001
Laboratory and imaging items	2.6 ± 0.5	1.0 ± 1.1	<0.0001
Suspected cholangitis (*n* = 30)			
Clinical items	1.1 ± 0.3	0.3 ± 0.5	<0.0001
Laboratory and imaging items	2.2 ± 0.8	1.1 ± 0.8	<0.0001
No cholangitis (*n* = 15)			
Clinical items	0.3 ± 0.7	0.2 ± 0.4	0.75
Laboratory and imaging items	1.9 ± 1.1	1.1 ± 0.8	0.03

Abbreviation: BARD, biliary atresia and related diseases.

### 3.3 Laboratory and imaging elements for the standardized BARD cholangitis definition

In 56/59 children (94.9%), at least one laboratory or radiological item (group B) was pathologic at cholangitis diagnosis. Findings are summarized in Table 1. Bile lakes at cholangitis diagnosis were only present in one patient 1/59 (1.7%). Positive blood cultures were also present in only one patient, 1/59 (1.7%).

In patients with confirmed cholangitis, 2.6 (±0.5) items from group B were present at cholangitis diagnosis, for the suspected cholangitis 2.2 (±0.8), and in cases of cholangitis diagnosis inconsistent with the standardized BARD definition 1.9 (±1.1) items (Table 2).

### 3.4 Correlation between clinical diagnosis and the standardized BARD cholangitis definition

The strongest correlation for clinical, laboratory, and imaging items with the suspected and confirmed cholangitis diagnosis according to the standardized BARD definition was seen for an increased or increasing GGT and/or bilirubin (*r* = 0.91; CI: 0.88–0.93) and a laboratory inflammatory response (*r* = 0.8; CI: 0.73–0.84) (Table S1).

### 3.5 Effect of treatment on the items of the standardized BARD cholangitis definition

After a 2‐ to 3‐week course of antibiotic treatment, the standardized BARD cholangitis definition was again applied, and a comparison was made with the pretreatment results, revealing a statistically significant reduction in the number of clinical, laboratory, and imaging items for both confirmed (*p* < 0.001) and suspected cholangitis (*p* < 0.001). In the group of patients with cholangitis not adhering to the BARD definition, a significant decrease was detectable for the laboratory and imaging items (*p* = 0.03), while no significant changes were present for the clinical items (*p* = 0.75) (Table 2).

In the group of confirmed cholangitis, antibiotic treatment was associated with significant changes in all group A and group B items (six out of seven), except for abdominal discomfort (*p* = 0.1). For suspected cholangitis, antibiotic treatment was associated with significant changes for three out of eight items: fever and/or shivering (*p* < 0.0001), the laboratory inflammatory response (*p* < 0.0001), and the GGT and/or bilirubin levels (*p* = 0.009). For patients with cholangitis not confirmed by the standardized BARD acute cholangitis definition, antibiotic treatment was associated with significant changes in the laboratory inflammatory response (*p* = 0.001), with a statistical trend for the GGT and/or bilirubin levels (*p* = 0.05), and only two out of seven diagnostic items responded to the treatment (Table 3).

**Table 3:** Detailed analysis of the effect of antibiotic treatment on clinical, laboratory and imaging items, stratified according to the standardized BARD cholangitis definition.

	Clinical items				Laboratory and imaging items			
	Fever and/or shivering	Stool color change	New/increasing jaundice	Abdominal discomfort^a^	Inflammatory response^b^	Increased/increasing transaminases	Increased/increasing GGT and/or bilirubin	Bile lakes
Confirmed cholangitis (*n* = 14)								
Pre‐Tt (*n*)	8 (57%)	11 (79%)	10 (71%)	6 (43%)	12 (86%)	11 (79%)	14 (100%)	0
Post‐Tt (*n*)	0	1	0	2	2	5	7	0
%‐change	100%	91%	100%	67%	83%	55%	50%	/
*p* Value	0.0003	<0.001	<0.001	0.10	<0.0001	0.02	0.01	/
Suspected cholangitis (*n* = 30)								
Pre‐Tt (*n*)	14 (47%)	7 (23%)	2 (7%)	9 (30%)	21 (70%)	19 (63%)	26 (87%)	1 (3%)
Post‐Tt (*n*)	0	5	1	3	0	15	17	2
%‐change	100%	29%	50%	67%	100%	21%	35%	50%
*p* Value	<0.0001	0.53	0.56	0.12	<0.0001	0.30	0.009	/
No cholangitis (*n* = 15)								
Pre‐Tt (*n*)	2 (13%)	1 (7%)	0	1 (7%)	7 (47%)	9 (60%)	12 (80%)	0
Post‐Tt (*n*)	2	2	1	0	0	9	7	0
%‐change	0	50%	100%	100%	100%	0%	42%	/
*p* Value	0.99	0.6	0.6	0.6	0.001	/	0.05	/

Abbreviations: BARD, biliary atresia and related diseases; GGT, gamma‐glutamyl transpeptidase; Tt, treatment.

^a^ Vomiting, poor feeding, irritability.

^b^ WBC and/or CRP and/or PCT.

## 4 DISCUSSION

Traditionally, cholangitis was diagnosed using the Charcot triad and later using the Tokyo guidelines.^12^, ^13^, ^14^ According to the first versions of those guidelines, definitive diagnosis of cholangitis requires confirmation of the biliary infection as the source of the illness with, for example, aspiration of purulent bile during Endoscopic Retrograde Cholangio Pancreatography.^15^, ^16^ Invasive methods were not included in the definition of cholangitis in the subsequent versions,^17^, ^18^ nevertheless, imaging criteria employed in adults (biliary dilatation, evidence of etiology on imaging [stricture, stone, stent, etc.]), are not useful in children with BA having undergone a HPE, and a “pediatric” definition for cholangitis in BA patients was needed. As there is no consensual diagnostic tool for acute cholangitis in this particular subset of patients, the convention was to base the diagnosis on the clinician's evaluation.

Consequently, the BARD network established the diagnostic criteria for cholangitis in children with BA within their first year after HPE, following a similar methodology as the Tokyo group. The standardized BARD cholangitis definition in BA patients after HPE is primarily based on the existing literature. Given the lack of evidence that mainly consists of retrospective cohort studies, a combination of “best available evidence” and an international expert consensus meeting allowed for agreement on a definition.^11^

An appraisal from clinicians is paramount to evaluate the validity of this new standardized BA associated cholangitis definition. In this study, we showed a strong correlation between clinicians' impression of cholangitis and the use of the standardized BARD cholangitis definition.

### 4.1 Clinical, laboratory, and imaging elements of the standardized BARD definition

Surprisingly, there was only a moderate association regarding the clinical elements, that is, fever or shivering, stool color changes with pale stools, increasing (macroscopic) jaundice, and abdominal discomfort. This and the fact that only 41% of patients believed by their physicians to have cholangitis had fever, might suggest that fever is not mandatory for the diagnosis of cholangitis in BA patients within the first year after HPE. Equally, increased or increasing transaminases exhibited also only a moderate correlation with the cholangitis appreciation. In future studies, the implementation of a more objective measure of the discolored stool by using the stool color card with a defined spectrum, ranging from 1−4 to 5−7 (http://www.basca.ch), could enhance the accuracy and consistency of reporting. This approach would provide a standardized framework for parents to assess and communicate stool color changes.

Two laboratory elements showed a very strong correlation between the standardized BARD cholangitis definition and the clinician's impression: increased or increasing GGT and/or bilirubin and laboratory inflammatory response.

In this cohort, the incidence of intrahepatic bile lakes and positive blood cultures was exceedingly low, raising the need for a prospective study to determine whether their inclusion in the definition is necessary.

### 4.2 Number of cholangitis items within the standardized BARD definition

For the confirmed cholangitis definition, the number of items defining cholangitis by the physician approached the standardized BARD definition. After applying the standardized BARD definition for suspected cholangitis patients, the clinical items identified by the physicians could be superimposed on the standardized BARD definition, but the latter had a lower laboratory and clinical threshold for defining suspected cholangitis when compared with clinical judgment. This is inherent with the fact that the term “suspected cholangitis” was not previously defined by physicians.

As for the group considered to have cholangitis by their physician and not by the standardized BARD definition, we observed that, notably, the clinical items were absent to meet the standardized definition diagnostic criteria. This suggests that the clinician strongly bases his/her decision on objective parameters such as laboratory and imaging, somehow ignoring the absence of clinical elements. This observation underscores the need for future studies from diverse centers to investigate further and refine diagnostic thresholds for cholangitis. Collaborative efforts across multiple institutions can provide a more comprehensive understanding of the diagnostic landscape and contribute to developing more nuanced and accurate criteria. These results show that in the future, the sensitivity, specificity, and positive and negative predictive value of the standardized BARD definition of cholangitis need to be assessed to allow for an accurate and universally applicable definition of BA associated cholangitis. This consensual tool will allow for comparisons between cohorts and potentially find modifiable factors for management improvement and ultimately improved prognosis of BA patients.

### 4.3 Pre and posttreatment use of the standardized BARD definition for cholangitis

In practice, as there is no gold standard for the definition of cholangitis, an indirect measure of the diagnosis and treatment efficacy is the treatment response, something which may now be better assessed using the new standardized cholangitis definition.

Indeed, for cases with confirmed cholangitis, all items, except abdominal discomfort, showed a significant decrease after treatment. Unquestionably, multiple factors can contribute to abdominal discomfort in BA patients, and it is thus not surprising that this parameter does not seem to respond to treatment in confirmed cholangitis patients.^19^

As for suspected cholangitis patients, the main parameters positively responding to treatment were fever and/or shivering, inflammatory response, and increased or increasing GGT and/or bilirubin. Further, these results raise the question of false positives: Whether patients with suspected cholangitis really had cholangitis or if the child was experiencing an unfavorable course of the disease seems debatable, as we had no gold standard for cholangitis diagnosis in this setting. Nevertheless, the response to treatment using the standardized definition posttreatment might represent a surrogate marker of cholangitis.

When analyzing patients treated as having cholangitis by their physicians but not meeting the standardized BARD definition, we observed that treatment seemed to impact inflammatory markers rather than clinical markers. This might suggest a gap in clinical evaluation that could potentially be overcome by utilizing the standardized BARD definition, which distinguishes between suspected and confirmed cholangitis.

Although this is the first study to evaluate the BARD cholangitis definition, it does have a few limitations. First, it is marred by the biases inherent to a retrospective, small cohort. A prospective study is being prepared to address this drawback. Second, the study is multicentric, and different management protocols were used (i.e., antibiotics, steroids). Nevertheless, the main surgical principles, postoperative care, and native liver survival were very similar^20^ (Calinescu AM, Rock NM, Wilde JCH et al., The Swiss national BA cohort: signs of a new age?; BARD Bruges, personal communication, 2022). Sensitivity and specificity analysis are problematic to perform on the cohort, mainly due to its retrospective nature and the lack of information on false positives and false negatives. To overcome this shortcoming, the future prospective study will aim to include all BA patients and have them monitored, at each clinical visit, with all items of groups A and B of the standardized definition, by well documenting also the clinician's appreciation, to allow for comparison. We thus aim to include patients initially suspected but later determined not to have cholangitis and also patients with a final cholangitis diagnosis that were not initially suspected. Furthermore, a prospective study is also required to assess further inclusion of bile lakes and positive blood cultures within the definition. Likewise, the forthcoming prospective study will address a potential selection bias linked to the fact that data collected during the decade before publication originate from two centers that actively contributed to the development of the BARD cholangitis definition.

To conclude, this study shows an encouraging, very strong correlation between the BARD standardized definition of post‐HPE cholangitis and the clinician's judgment. A prospective study to further refine this standardized definition of cholangitis in BA patients will be needed, with the ultimate goal to improve the prognosis of these vulnerable patients.

## CONFLICT OF INTEREST STATEMENT

The authors declare no conflict of interest.

## ETHICS STATEMENT

The present study follows the guidelines of the revised UN Declaration of Helsinki in 1975 and its latest amendment in 2013 (7th revision) and was approved by the Local Research Ethical Committee at the Hannover Medical School (no. 41/2000) and complies with the local regulations in the Geneva University Hospitals, Swiss Ethics (no. 06‐050). Informed consent was obtained from each patient's legal guardian.

## Supporting information

Supporting information.
