# Supplementary material for: Retrospective analysis of the standardized BARD criteria for acute cholangitis in biliary atresia patients
Source: JPGN Rep. 2024 Apr 12;5(3):309–16. doi: 10.1002/jpr3.12071 (PMC11322016; doi:10.1002/jpr3.12071)
Supplement: Supplementary file 1 — Supporting information. [file JPR3-5-309-s001.docx]

Supplementary material

**Table S1.** Correlation between elements at cholangitis diagnosis and the Standardized BARD cholangitis definition

| **Elements at cholangitis diagnosis (n=59)** |  | **Correlation between elements at cholangitis diagnosis and Standardized BARD definition** | |
| --- | --- | --- | --- |
|  | n | r | CI |
| **Clinical elements** |  |  |  |
| Fever and/or shivering | 24 | 0.6 | 0.50 – 0.69 |
| Stool color change | 19 | 0.58 | 0.47 – 0.67 |
| New/increasing jaundice | 12 | 0.49 | 0.37 – 0.6 |
| Abdominal discomfort | 16 | 0.49 | 0.37 – 0.6 |
| **Laboratory and imaging elements** |  |  |  |
| Inflammatory response | 41 | **0.8** | **0.73 – 0.84** |
| Increased/increasing transaminases | 40 | 0.76 | 0.69 – 0.81 |
| Increased/increasing GGT and/or bilirubin | 53 | **0.91** | **0.88 – 0.93** |
| Bile lakes | 1 | 0.1 | 0.04 – 0.3 |
